# Tylosin Removal Under Pb^2+^ Stress Using *Kurthia gibsonii* TYL-A1: Redox Adaptation and Transcriptomic Insights

**DOI:** 10.3390/antiox15080958

**Published:** 2026-07-31

**Authors:** Ye Wang, Heshi Tian, Xiqing Zhang, Yuanquan Zhu, Lingcong Kong, Changlong Gou, Hongxia Ma, Xiuzhen Yu, Wenyan Yang, Yunhang Gao

**Affiliations:** 1 College of Veterinary Medicine, Jilin Agricultural University, Changchun 130118, China; 2 Northeast Agricultural Research Center of China, Jilin Academy of Agricultural Sciences, Changchun 130033, China; 3 College of Animal Science and Technology, Inner Mongolia Minzu University, Tongliao 028000, China; 4 Institute of Agricultural Equipment, Xinjiang Academy of Agricultural Sciences, Urumqi 830091, China

**Keywords:** TYL, removal, oxidative stress, antibiotic–heavy metal co-contamination

## Abstract

With the increasing prevalence of antibiotic–heavy metal co-contamination in agricultural wastewater, elucidating the antibiotic removal capacity of degrading bacteria under heavy metal stress, as well as their adaptive mechanisms, is of considerable theoretical significance and practical value. In this study, the highly efficient TYL-degrading strain TYL-A1 was selected as a model organism, and the effects of Pb^2+^ exposure on its TYL removal performance, oxidative stress response, cell surface characteristics, and transcriptional regulation were systematically investigated. The results showed that TYL removal remained above 85% after 72 h under a nominal Pb^2+^ concentration of 100 mg/L in the phosphate-containing MSM system. The phosphate may have reduced Pb^2+^ bioavailability. Nominal Pb^2+^ exposure increased the activities of superoxide dismutase (SOD) and catalase (CAT), accompanied by elevated levels of reactive oxygen species (ROS) and malondialdehyde (MDA), as well as a decrease in adenosine triphosphate (ATP) content. This indicates that the strain experienced pronounced oxidative stress and altered energy metabolism. Meanwhile, cell membrane permeability increased. Scanning electron microscopy (SEM) and Fourier-transform infrared spectroscopy (FTIR) analyses showed that the overall cellular morphology remained intact, whereas the membrane surface structure and related cellular components underwent adaptive changes. According to a transcriptomic analysis, the differentially expressed genes were mainly enriched in pathways associated with primary metabolism, transmembrane transport, ABC transporters, and two-component systems. In summary, strain TYL-A1 maintained high TYL removal performance under the tested nominal Pb^2+^ concentrations in the present phosphate-containing culture system, suggesting its potential applicability in the bioremediation of water bodies co-contaminated with antibiotics and heavy metals.

## 1. Introduction

In intensive livestock and poultry farming systems, the long-term and continuous use of veterinary antibiotics has not only safeguarded animal health and improved production efficiency but has also created significant environmental risks associated with antibiotic residues [1,2,3]. Some antibiotics and their metabolites cannot be fully absorbed or metabolized by animals and are therefore frequently released into soils and aquatic environments through manure and wastewater [4,5,6]. Upon their introduction into the environment, they may migrate, accumulate, and persist over extended periods, thereby disrupting microbial community structure and key ecological processes and posing potential risks to public health [7,8]. Tylosin (TYL) is a typical macrolide veterinary antibiotic. Its widespread use, complex environmental behavior, and potential risk of inducing antibiotic resistance have made it a key pollutant which requires close attention in pollution control efforts in the livestock and poultry farming context [9,10,11].

Microbial removal is considered an important bioremediation approach for reducing environmental TYL residues and associated ecological risks. This process depends on bacterial growth and metabolism, pollutant uptake and transport, and intracellular and extracellular enzyme systems involved in degradation. Through biotransformation reactions such as oxidation, reduction, and hydrolysis, microorganisms can gradually transform TYL into less toxic intermediates, thereby reducing its environmental risk [12]. However, the effectiveness of bioremediation is highly sensitive to environmental conditions, and microbial degradation efficiency is typically influenced by multiple abiotic and biotic factors, including temperature, pH, nutrient availability, pollutant bioavailability, and microbial community interactions [13]. Therefore, in complex contaminated environments, it has yet to be determined whether TYL-degrading bacteria can consistently maintain their degradation capacity, and this has limited their practical application.

Co-contamination by antibiotics and heavy metals is a common form of complex pollution in livestock, poultry, and agricultural environments. Combined exposure to these pollutants may result in additive or synergistic toxic effects, making remediation more difficult and increasing ecological risks [14,15]. Pb^2+^, a typical highly toxic heavy metal pollutant, is characterized by non-biodegradability, environmental persistence, and strong biological toxicity [16]. In scenarios such as aquaculture wastewater discharge and farmland manure application, TYL residues may coexist with Pb^2+^ and impose combined stress on environmental microorganisms. Pb^2+^ stress can induce ROS accumulation and disrupt the dynamic balance between ROS production and antioxidant scavenging, thereby triggering oxidative damage, including lipid peroxidation, enzyme inactivation, and DNA damage [17,18,19]. These processes may further impair cell membrane homeostasis, energy metabolism, and transmembrane transport. To counteract ROS-mediated cytotoxicity, microorganisms typically activate antioxidant enzyme systems, such as SOD and CAT, to scavenge superoxide anions and H_2_O_2_ and maintain redox homeostasis. These stress responses may further affect TYL uptake and metabolism, as well as the expression of genes encoding enzymes involved in TYL degradation.

Although various TYL-degrading microorganisms have been reported, previous studies have largely focused on degradation efficiency, degradation products, and strain screening under conditions involving a single antibiotic [20,21]. There is still a lack of systematic understanding of how TYL-degrading bacteria coordinate metal tolerance, oxidative stress defense, membrane homeostasis maintenance, and the expression of degradation functions under heavy metal stress. This study focused on the highly efficient, previously isolated TYL-degrading strain *Kurthia gibsonii* TYL-A1 [22], and aimed to systematically evaluate its TYL removal performance under Pb^2+^ stress. By analyzing indicators such as ROS production, MDA accumulation, SOD and CAT activities, membrane-related characteristics, and ATP levels, this study elucidated the strain’s oxidative stress response and the mechanisms underlying the maintenance of its degradation capacity. Through transcriptomic analysis, this study investigated the molecular responses of TYL-A1 related to redox regulation, membrane transport, energy metabolism, and potential degradation-associated pathways under combined TYL–Pb^2+^ stress and further evaluated its application potential in aquaculture wastewater. These findings provide a theoretical basis for understanding how TYL-A1 maintains TYL removal under Pb^2+^ induced oxidative stress and for the reliable application of functional pollutant-degrading bacteria in antibiotic–heavy metal co-contaminated environments.

## 2. Materials and Methods

### 2.1. Materials

TYL-A1 is a highly efficient TYL-degrading strain that was isolated and preserved by our research group and cultured in TSB medium [22]. The HPLC-grade TYL standard was purchased from Yuanye Biotechnology Co., Ltd. (Shanghai, China), and the Pb^2+^ standard solution was obtained from Solarbio Life Sciences (Beijing, China). HPLC-grade methanol and acetonitrile were acquired from Thermo Fisher Scientific Co., Ltd. (Waltham, MA, USA). HPLC-grade formic acid was sourced from Fuchen Chemical Reagents (Tianjin, China). The mineral salt medium (MSM) was prepared as described in previous studies [23]. Unless otherwise specified, all reagents and chemicals were of analytical grade.

### 2.2. Determination of TYL

Strain TYL-A1 was cultured in TSB medium until it reached the logarithmic phase, harvested by centrifugation, washed twice with PBS, and resuspended to an OD_600_ of 2.0. The suspension was inoculated into MSM at a 3% (*v*/*v*) inoculum. Unless otherwise stated, an initial TYL concentration of 75 mg/L was used throughout this study. All treatments were performed in triplicate and incubated in the dark at 30 °C and 130 r/min. The TYL concentration was determined by HPLC-UV as previously described [22]. Separation was achieved on a ZORBAX Eclipse Plus C18 column (250 mm × 4.6 mm, 5 μm). The mobile phase consisted of acetonitrile and 0.1% aqueous formic acid (67:33, *v*/*v*) at a flow rate of 0.6 mL/min. The column temperature, detection wavelength, injection volume, and retention time were 40 °C, 285 nm, 25 μL, and 16 min, respectively. Prior to analysis, samples were centrifuged and filtered through 0.22 μm membrane filters. In this study, the main experimental groups were defined as follows: Control, TYL-A1 and TYL; A, TYL-A1 alone; APb, TYL-A1 + Pb^2+^; and APbT, TYL-A1 + Pb^2+^ + TYL.

TYL removal efficiency (%) = (T0 − T1)/T0 × 100

where T0 represents the initial concentration of TYL (mg/L) and T1 represents the residual concentration of TYL (mg/L).

### 2.3. Effects of Metal Ions and Pb^2+^ Concentrations on TYL Removal

To evaluate the effects of metal ions on TYL removal, strain TYL-A1 was inoculated into MSM containing TYL and various metal ions (Cu^2+^, Mn^2+^, Fe^3+^, Fe^2+^, Zn^2+^, Ca^2+^, Ni^2+^, Cd^2+^, and Pb^2+^, pH 7.0), each at a final concentration of 10 mg/L. Samples were collected every 24 h to determine residual TYL concentrations via HPLC. Then, TYL-A1 was exposed to nominal Pb^2+^ concentrations of 0, 10, 50, 100, and 200 mg/L and the effects were assessed. Samples were collected every 24 h to determine the residual TYL concentrations via HPLC and culture turbidity was determined by measuring the OD_600_. The OD_600_ values of each treatment were corrected by subtracting the corresponding values of sterile controls. Abiotic and heat-inactivated cell controls were included to distinguish live cell-associated TYL removal from abiotic loss and passive adsorption. The concentration of filterable lead in the supernatant was determined using inductively coupled plasma–mass spectrometry (ICP-MS). After incubation, the cultures were centrifuged to remove bacterial cells and large particulate matter. The resulting supernatant was filtered through a 0.45 μm aqueous membrane filter, acidified with high-purity nitric acid, and stored at 4 °C prior to analysis. Lead concentrations were determined using an ICP-MS instrument (Agilent Technologies, Inc., Santa Clara, CA, USA) and quantified using external calibration. Procedural blanks and quality control samples were included throughout the analytical process. However, no filtration-specific spike-recovery tests or solid-phase characterization were performed. Therefore, the comparison provided only an operational distinction between filterable and non-filterable Pb and could not quantitatively distinguish adsorption from precipitation.

### 2.4. Oxidative Stress, Antioxidant Enzymes, and ATP Assays

TYL-A1 cells were collected at different time points after exposure to Pb^2+^, TYL, or Pb^2+^ + TYL for the determination of physiological and biochemical parameters. Intracellular ROS levels were measured using the DCFH-DA staining method. Briefly, the bacterial suspension was adjusted to an OD_600_ of 0.5, supplemented with DCFH-DA at a final concentration of 10 μM, and incubated at 37 °C in the dark for 30 min. The fluorescence intensity was then measured using a fluorescence microplate reader (Thermo Fisher Scientific, Waltham, MA, USA) at an excitation wavelength of 488 nm and an emission wavelength of 525 nm. For biochemical assays, the collected cells were washed with PBS and sonicated in an ice bath. MDA content was determined using an MDA assay kit (Beyotime Biotechnology, Shanghai, China). The sonicated samples were centrifuged at 12,000× *g* for 30 min, the supernatants were collected, and the ATP levels, SOD activity, and CAT level were measured according to the manufacturers’ instructions. The total protein concentration was determined using a BCA protein assay kit and used to normalize ATP levels, MDA content, SOD activity, and CAT activity.

### 2.5. Determination of Cell Surface Properties of Strain TYL-A1

TYL-A1 cells exposed to Pb^2+^ or Pb^2+^ + TYL at different time points were collected and resuspended in PBS. The bacterial suspension (500 μL) was mixed with 100 μL of 0.1 mg/mL crystal violet and incubated at 37 °C for 10 min. After centrifugation at 8000 r/min for 10 min, the absorbance of the supernatant was measured at 590 nm. Crystal violet adsorption was calculated as follows:

Crystal violet adsorption (%) = (OD_0_ − OD_1_)/OD_0_ × 100

where OD_0_ is the initial OD_590_ value of the crystal violet solution and OD_1_ is the OD_590_ value of the supernatant from the experimental group after incubation with bacterial cells.

Cell surface hydrophobicity (CSH) was determined using the MATH method [24]. TYL-A1 cells exposed to Pb^2+^ or Pb^2+^ + TYL at different time points were collected, resuspended in PBS, and adjusted to an OD_600_ of 0.6. The bacterial suspension was mixed with 1.5 mL of xylene, vortexed, and left undisturbed for 60 min. The absorbance of the lower aqueous phase was then measured at 600 nm. CSH was calculated as follows:

CSH(%) = 100 × (A_0_ − A_1_)/A_0_

where A_0_ represents the initial OD_600_ value of the aqueous phase and A_1_ represents the final OD_600_ value of the aqueous phase after standing and separation.

### 2.6. Cell Morphology and Surface Characterization

TYL-A1 cells were cultured in MSM under control, Pb^2+^, and Pb^2+^ + TYL conditions at 30 °C and 130 r/min in the dark for 24 h. For SEM analysis (Sigma, Jena, Germany), cells were collected, washed three times with PBS, fixed with glutaraldehyde for 24 h, dehydrated through a graded ethanol series, and observed using a scanning electron microscope. For FTIR analysis, cells exposed to Pb^2+^ or Pb^2+^ + TYL were collected, dried, mixed with anhydrous KBr, pressed into pellets under 6–8 MPa, and analyzed by FTIR (Thermo Fisher Scientific, Waltham, MA, USA) to assess changes in surface functional groups.

### 2.7. Transcriptome Analysis

For transcriptome analysis, TYL-A1 cells from different treatment groups were collected after 24 h of incubation. Total RNA was extracted, rRNA was removed, and sequencing libraries were constructed and sequenced on an Illumina platform. After quality control, clean reads were used for gene expression quantification. Differentially expressed genes (DEGs) were identified using padj < 0.05 and |log_2_FC| ≥ 1 as the criteria. GO and KEGG enrichment analyses were performed using ClusterProfiler and the sequencing data were deposited under accession number PRJNA1457065.

### 2.8. Real-Time Quantitative PCR (RT-qPCR)

Primers were designed using SnapGene software (version 6.0.2) and synthesized by Sangon Biotech Co., Ltd. (Shanghai, China). qPCR amplification was performed using the TB Green^®^ Premix Ex Taq™ II kit. Relative gene expression levels were normalized to the 16S rRNA gene. Amplification was conducted using the LightCycler^®^ 96 real-time PCR system (Roche, Basel, Switzerland), and RT-qPCR data were analyzed using a comparative cycle threshold method, 2^−ΔΔCt^, to determine the relative fold changes in gene expression. Transcriptomic analysis identified *GM002829*, *GM001788*, *GM000582*, *GM001387*, and *GM001392* as key candidate genes that may potentially be associated with the TYL removal process and their expression levels were further validated via quantitative RT-qPCR.

### 2.9. Application in Simulated Farm Wastewater

Aquaculture wastewater was settled for 24 h, filtered through a 0.45 μm membrane (pH 7.0), diluted 20-fold, and sterilized at 121 °C for 20 min. The experiment included three groups: a blank control, TYL-A1 treatment, and Pb^2+^ + TYL stress treatment. Each treatment was performed in triplicate. Samples were collected every 24 h to determine the TYL concentration. The chemical oxygen demand (COD), nitrate nitrogen (NO_3_^−^-N), total nitrogen (TN), and total phosphorus (TP) were also measured.

### 2.10. Statistical Analysis

Statistical analyses and graph generation were performed using GraphPad Prism 10.0 (GraphPad Software, Inc., San Diego, CA, USA). All experiments were conducted with three biological replicates. The data are presented as the mean ± standard deviation. Statistical comparisons were performed using Student’s t-test or two-way analysis of variance (ANOVA), as appropriate. The asterisk (*) denotes statistically significant differences compared with the control group (*p* < 0.05).

## 3. Results and Discussion

### 3.1. Effects of Metal Ions on TYL Removal Using Strain TYL-A1

**Figure 1:**
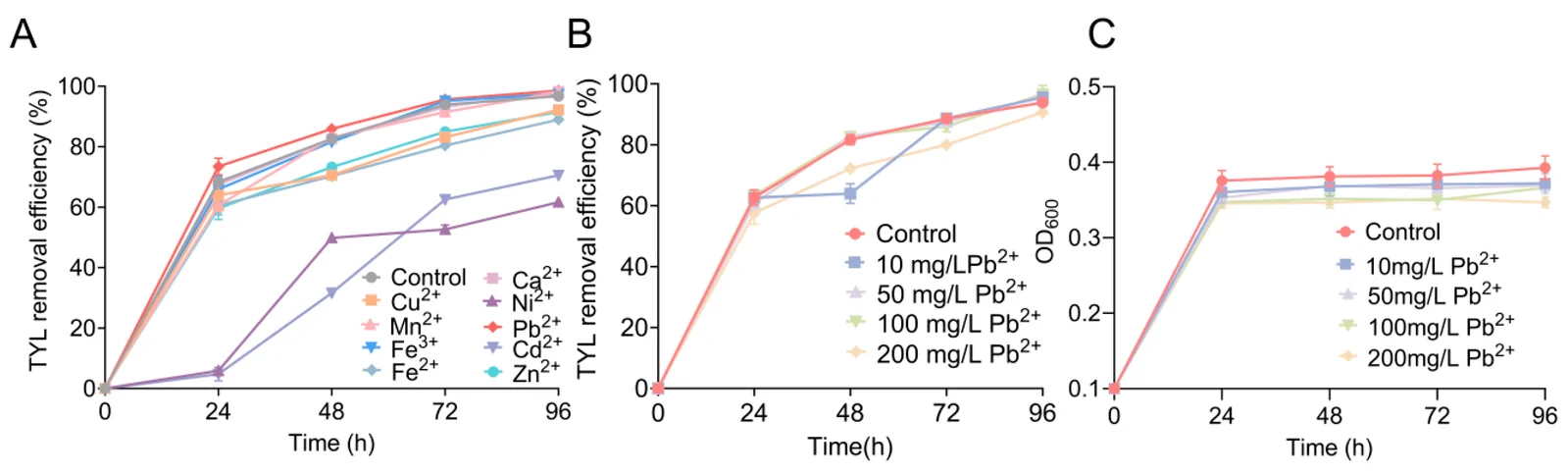
The effects of metal ions and Pb^2+^ exposure on TYL removal using strain TYL-A1 and the associated OD_600_ measurements. (**A**) The effect of different metal ions on the removal rate of TYL; (**B**) TYL removal at different nominal Pb^2+^ concentrations; (**C**) the growth of strain TYL-A1 under different concentrations of Pb^2+^ stress (OD_600_).

As shown in Figure 1A, the tested metal ions affected the TYL removal capabilities of strain TYL-A1 to different extents. Several metal ions reduced TYL removal under the tested conditions, whereas Pb^2+^ showed a relatively limited inhibitory effect. When TYL-A1 was exposed to nominal Pb^2+^ concentrations of 0–200 mg/L, TYL removal was generally maintained, although a slight decrease was observed at 200 mg/L (Figure 1B). Under the present MSM conditions, a nominal Pb^2+^ addition of 100 mg/L remained above a TYL removal rate of 85% after 72 h. Meanwhile, OD_600_-based biomass exhibited no significant difference from the control group (Figure 1C), suggesting that this level of Pb^2+^ addition did not markedly reduce bacterial biomass. Therefore, a nominal Pb^2+^ addition of 100 mg/L was selected for subsequent experiments as a non-inhibitory stress condition to investigate the physiological and molecular responses of TYL-A1 under Pb^2+^ exposure. As shown in Table S1, TYL loss in the abiotic and heat-inactivated cell controls was much lower than that in the live-cell treatment. Therefore, live TYL-A1 cells played a major role in TYL removal, although adsorption and abiotic loss could not be completely excluded. Under this condition, the filterable Pb^2+^ concentrations in the filtered supernatants for all treatments were much lower than the initial nominal concentration, and bacterial treatment further reduced the residual filterable Pb^2+^ level (Table S1). TYL itself may also affect the partitioning or chemical speciation of Pb^2+^ in the MSM system. It should be noted that, in phosphate-containing environments, Pb^2+^ may bind with phosphate, colloids, or organic matter or form precipitates [25,26]. Therefore, the Pb^2+^ concentrations measured in this study represent the Pb^2+^ fraction that remains in the supernatant after centrifugation and 0.45 μm filtration. This indicator can be used to evaluate the effects of different treatments on residual filterable Pb levels and potential Pb^2+^ mobility.

### 3.2. Antioxidant Stress Response Capacity

**Figure 2:**
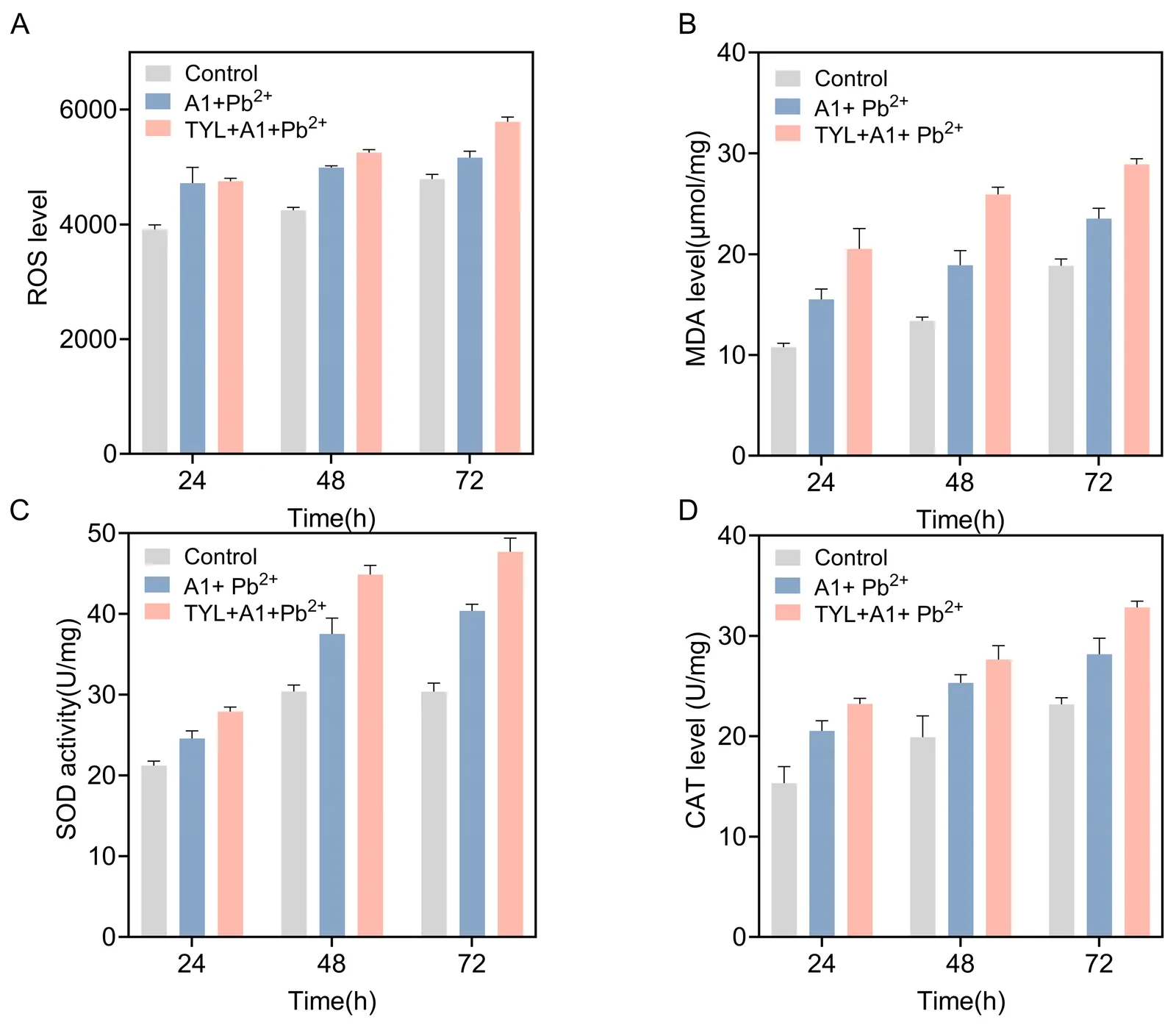
Changes in oxidative stress-related indicators of strain TYL-A1 under Pb^2+^ stress. (**A**) ROS level; (**B**) MDA level; (**C**) SOD activity; (**D**) CAT level.

Compared with the control group, intracellular ROS levels in the Pb^2+^ and Pb^2+^ + TYL treatment groups gradually increased with prolonged exposure (Figure 2A), which was indicative of sustained oxidative pressure in TYL-A1 cells. In addition to causing oxidative damage, ROS may also act as redox signals that are involved in the activation of cellular defense responses. MDA is a commonly used marker of membrane lipid peroxidation. As shown in Figure 2B, the MDA levels in the exposed bacterial cells were higher than those in the control group, indicating that exposure to Pb^2+^ or Pb^2+^ + TYL disrupted cellular redox homeostasis and induced oxidative damage to membrane lipids. SOD and CAT activities increased under Pb^2+^ and Pb^2+^ + TYL exposure (Figure 2C,D), reflecting activation of a compensatory antioxidant response. SOD converts superoxide anions into H_2_O_2_, whereas CAT subsequently decomposes H_2_O_2_, thereby helping to reduce the intracellular oxidative burden. Nevertheless, this antioxidant response was insufficient to fully restore redox homeostasis. ROS continued to accumulate with prolonged exposure, while MDA levels remained elevated, indicating that ROS generation and oxidative damage accumulation exceeded the antioxidant scavenging and cellular repair capacity of TYL-A1. The concurrent decline in intracellular ATP may have further constrained energy-dependent repair and membrane-maintenance processes. Thus, although the increased SOD and CAT activities may have alleviated part of the oxidative burden, they were unable to completely counteract the sustained oxidative pressure during prolonged exposure. This pattern is consistent with previous studies showing that heavy metals and antibiotics can induce persistent oxidative stress in microorganisms [27,28,29,30]. The concurrent increases in ROS and MDA levels, together with the compensatory activation of SOD and CAT, suggest that Pb^2+^-associated oxidative stress contributed to membrane lipid peroxidation and may have further affected membrane permeability and cellular homeostasis in strain TYL-A1.

### 3.3. Intracellular ATP Levels Under Pb^2+^ and Pb^2+^ + TYL Stress

**Figure 3:**
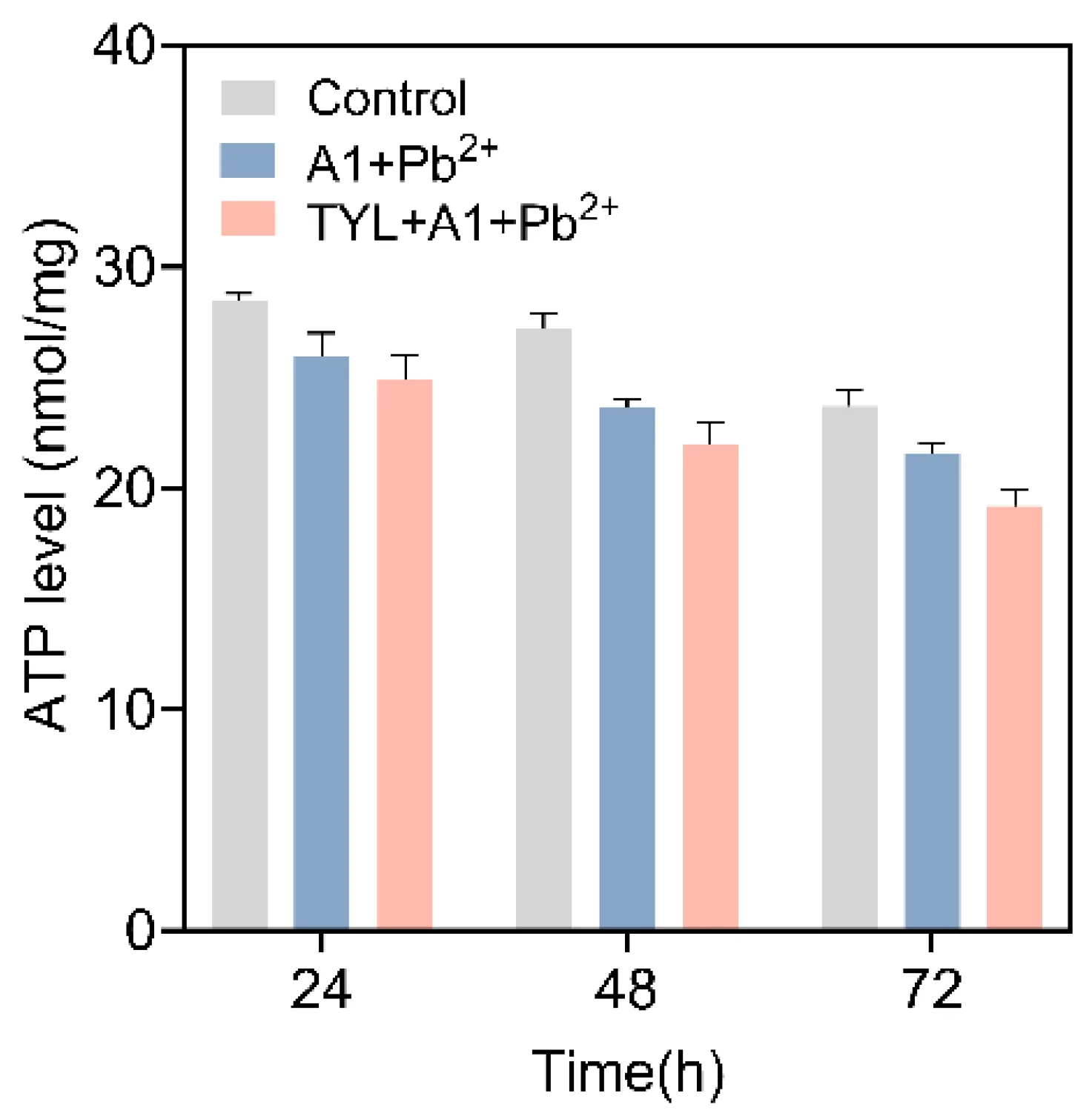
Changes in intracellular ATP levels of strain TYL-A1 under Pb^2+^ stress.

ATP is an important energy molecule required for maintaining fundamental cellular activities and is involved in processes such as metabolism, solute transport, DNA replication, protein synthesis, and responses to environmental stress [31,32]. As shown in Figure 3, compared with the control group, both Pb^2+^ and Pb^2+^ + TYL treatments led to a decrease in intracellular ATP levels, indicating that Pb^2+^ exposure may interfere with energy metabolism in TYL-A1 and reduce the supply of energy available for growth, substrate transport, detoxification, and defense responses. Therefore, under Pb^2+^ stress or combined Pb^2+^ + TYL stress, TYL-A1 may be unable to maintain a fully normal metabolic state. Instead, the changes in membrane-related indicators and stress responses suggest that TYL-A1 may adjust its energy utilization strategy to maintain a certain level of TYL removal capacity under energy-limited conditions.

Consistent with the decline in ATP levels, ribosome-related pathways also showed marked changes. Ribosomes are responsible for protein synthesis, and ribosomal regulation is an important mechanism by which bacteria adapt to environmental stress and maintain cellular homeostasis [33,34]. As a macrolide antibiotic, TYL primarily acts on the bacterial 50S ribosomal subunit and interferes with protein synthesis, thereby imposing additional pressure on translation-related processes. When exposed to Pb^2+^ stress alone, ribosome-related genes were generally upregulated, suggesting that cells may enhance protein synthesis to meet the demands of stress responses and damage repair. However, under combined Pb^2+^ and TYL stress, ribosome-related differentially expressed genes were predominantly downregulated, which indicates that combined stress may suppress translational activity and thereby reduce the energy expenditure associated with protein synthesis [35]. This change is consistent with the mechanism by which TYL targets the bacterial protein synthesis system, suggesting that under dual stress, TYL-A1 may downregulate certain translational processes to alleviate the energy burden and redirect resources toward more critical adaptive processes, such as the maintenance of membrane homeostasis, transport regulation, detoxification, and stress defense [36]. Therefore, the decrease in ATP levels and the transcriptional regulation of ribosome-related genes indicate that TYL-A1 maintained TYL removal under Pb^2+^-associated stress not through an overall enhancement of metabolic activity, but likely through energy reallocation and adaptive regulation of translation-related processes [37].

### 3.4. Effects of Pb^2+^ or Pb^2+^ + TYL Exposure on the Cell Surface Physicochemical Properties of TYL-A1

**Figure 4:**
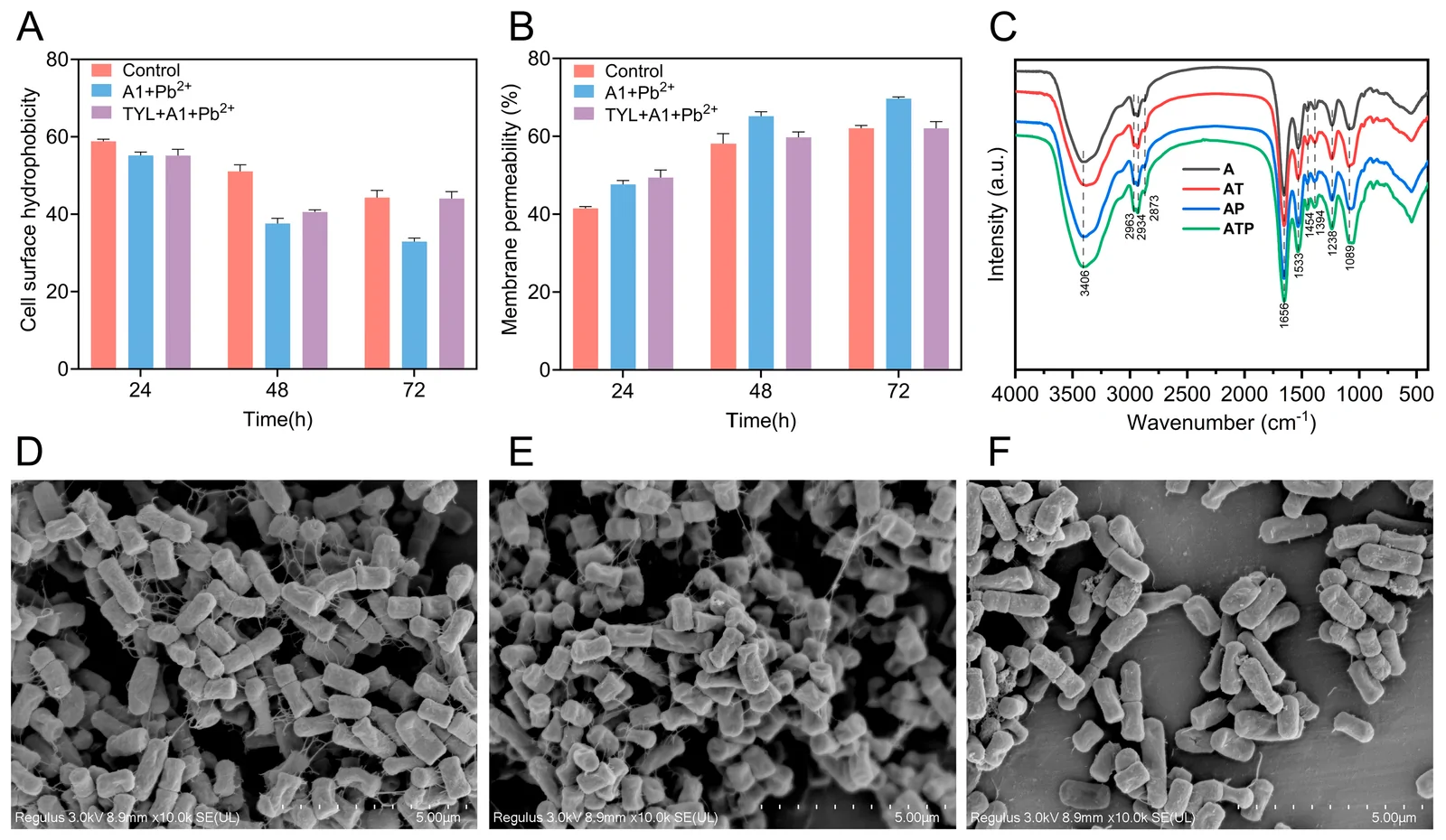
Cell surface characteristics and morphological changes in strain TYL-A1 under Pb^2+^ and combined Pb^2+^ + TYL stress. (**A**) CSH; (**B**) membrane permeability; (**C**) FTIR spectra; (**D**) SEM images of the control group; (**E**) SEM images of cells exposed to Pb^2+^; (**F**) SEM images of combined Pb^2+^ + TYL treatment.

The bacterial cell membrane is a key barrier for maintaining cellular integrity and responding to environmental stress [38]. Compared with the control group (Figure 4B), cell membrane permeability in the Pb^2+^ and Pb^2+^ + TYL treatment groups gradually increased with prolonged exposure time, indicating that Pb^2+^ or Pb^2+^ + TYL exposure may alter the membrane integrity or physiological state of TYL-A1. This change may reflect cell membrane damage. It may also be associated with adaptive regulation by bacterial cells in response to external stress.

CSH is an important factor influencing the contact and nonspecific adsorption between bacterial cells and organic pollutants and may further affect pollutant bioavailability and degradation processes [39]. In this study, exposure to Pb^2+^ or Pb^2+^ + TYL decreased the CSH of TYL-A1 (Figure 4A), suggesting that the physicochemical properties of the bacterial surface were altered, which may affect the contact, adsorption, and bioavailability of TYL or other organic pollutants. The decrease at 48 h followed by a rebound at 72 h in the TYL-A1 + Pb^2+^ + TYL treatment group may indicate a time-dependent adaptive response in the cell surface properties of TYL-A1 under combined Pb^2+^ and TYL exposure. At 48 h, the simultaneous presence of TYL and Pb^2+^ may have disrupted the cell membrane and surface structures, resulting in alterations in the composition of cell-surface lipids, proteins, or extracellular polymeric substances (EPSs), together with greater exposure of hydrophilic groups. These changes may have contributed to the reduction in cell surface hydrophobicity. With prolonged exposure to 72 h, TYL-A1 may have gradually adapted to the combined stress by remodeling the cell envelope, adjusting membrane lipid composition, modifying EPS secretion, or regulating the expression of surface proteins, thereby leading to a partial recovery of cell surface hydrophobicity. Similar increases in CSH and membrane permeability under Pb^2+^ exposure have been reported in *Vibrio cholerae* [40], indicating that changes in surface physicochemical properties may be a common bacterial response to heavy metal stress.

Changes in the surface functional groups of TYL-A1 were analyzed using FTIR. As shown in Figure 4C, the broad peak at 3406 cm^−1^ corresponds to the stretching vibration of -OH; the absorption peaks at 2963 cm^−1^, 2934 cm^−1^, and 2873 cm^−1^ are attributed to the asymmetric and symmetric stretching vibrations of C-H in saturated hydrocarbons; the peaks at 1656 cm^−1^ and 1533 cm^−1^ correspond to the C=O stretching vibration of amide I and the N-H bending vibration of amide II, respectively; the peaks at 1454 cm^−1^ and 1394 cm^−1^ are assigned to C-H bending vibrations; the peak at 1238 cm^−1^ is attributed to C-N stretching vibration; and the peak at 1089 cm^−1^ corresponds to C-O stretching vibration. The positions of the main characteristic peaks in the FTIR spectra did not shift markedly, indicating that exposure to Pb^2+^ and Pb^2+^ + TYL did not induce significant alterations in the types of major functional groups on the cell surface. However, changes in the intensity of some peaks suggested that the relative contents of surface components such as proteins, polysaccharides, or lipids may have been altered. Further observations of bacterial morphology revealed that TYL-A1 exhibited certain morphological differences under different treatment conditions. Neither Pb^2+^ treatment (Figure 4E) nor Pb^2+^ + TYL treatment (Figure 4F) caused obvious cell rupture or severe morphological damage. However, compared with the control group (Figure 4D), the treated cells showed changes in surface attachments and cell aggregation patterns, indicating that Pb^2+^ stress may affect the cell surface structure or the distribution of extracellular polymeric substances [41]. Such changes may help to reduce direct metal toxicity by enhancing extracellular binding or surface-associated sequestration of Pb^2+^ [42].

The increases in membrane permeability and CSH, together with FTIR peak intensity changes and altered cell surface morphology, indicate that TYL-A1 underwent membrane and surface remodeling under Pb^2+^-associated stress. Combined with the observed ROS and MDA accumulation, these membrane changes were likely associated with oxidative damage. Meanwhile, when ATP availability is reduced, surface remodeling and permeability adjustments may help the cells to maintain substrate contact, transport regulation, and TYL removal rather than relying on an overall enhancement of metabolism. Therefore, the membrane response of TYL-A1 appears to involve both stress-induced damage and adaptive regulation, which may contribute to its ability to maintain TYL removal under combined Pb^2+^ + TYL stress.

### 3.5. Transcriptomic Responses of TYL-A1 Under Pb^2+^ and Pb^2+^ + TYL Stress

To further reveal the molecular mechanisms by which TYL-A1 survives and adapts to co-contamination stress, transcriptomic analysis was performed to characterize the response of TYL-A1 to the stress conditions. DEGs in each group were identified using |log_2_FC| ≥ 1 and padj < 0.05 as the selection criteria. The results (Figure S2) showed that 653, 422, and 95 differentially expressed genes were identified in the comparisons of AP vs. A, APbT vs. A, and APbT vs. APb, respectively. Pb^2+^ stress alone induced the largest number of DEGs, indicating that under the tested conditions, nominal Pb^2+^ exposure was the major factor shaping the transcriptional response of TYL-A1. In contrast, the addition of TYL under Pb^2+^ background conditions generated only 95 DEGs, suggesting that the additional transcriptional perturbation caused by TYL was relatively limited and mainly involved processes related to transport, signal regulation, and metabolic adaptation.

**Figure 5:**
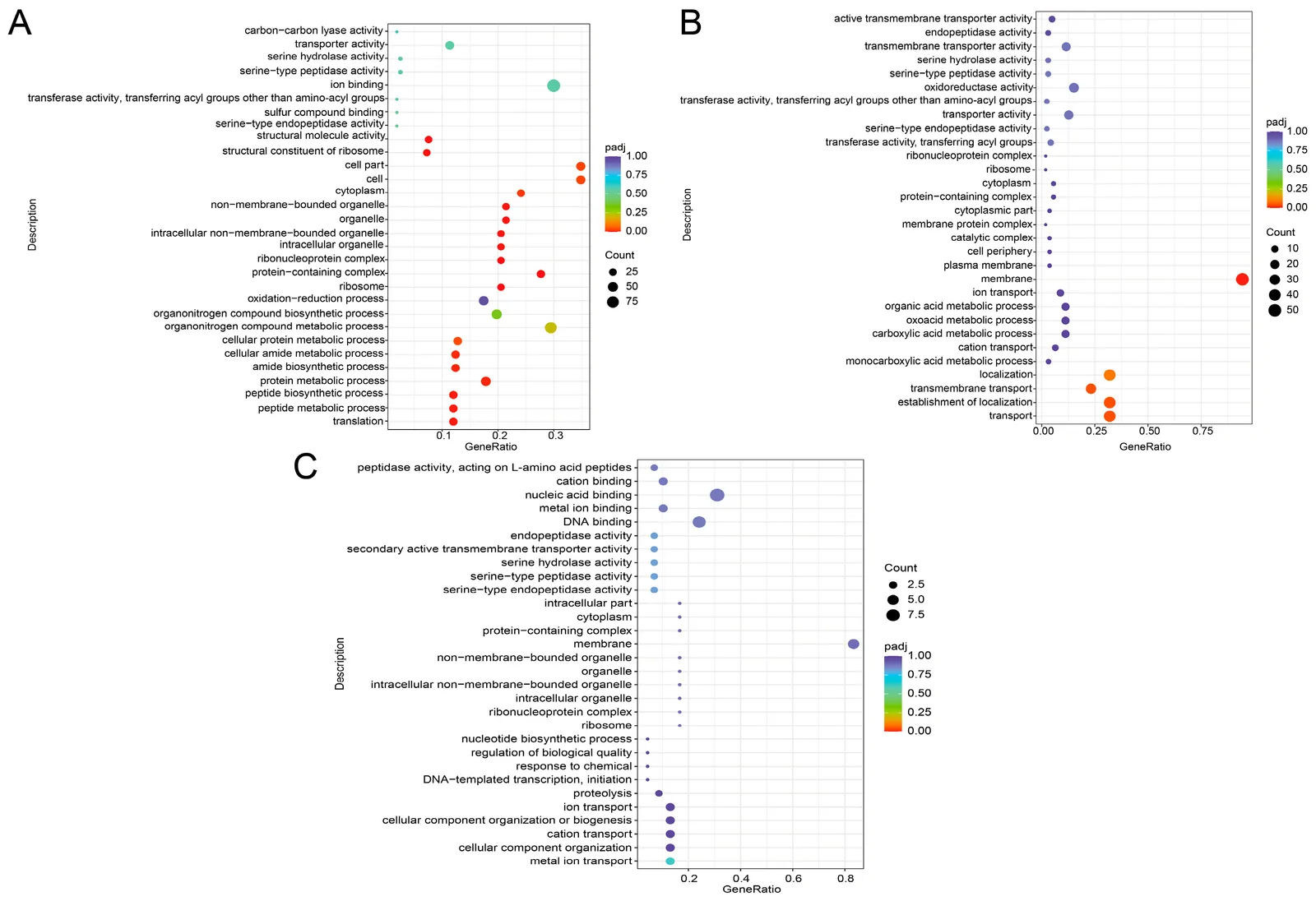
GO functional enrichment analysis of differentially expressed genes. (**A**) AP vs. A; (**B**) APbT vs. A; (**C**) APbT vs. APb.

GO enrichment analysis was performed to further characterize the functional categories affected by Pb^2+^ and Pb^2+^ + TYL stress. The enriched GO terms were mainly associated with ribosome-related functions, translation, peptide and protein metabolic processes, membrane components, transmembrane transport, ion transport, metal ion binding, oxidoreductase activity, and organic acid metabolism. In the AP vs. A comparison (Figure 5A), the enriched terms were mainly related to translation, ribosome, ribonucleoprotein complexes, peptide biosynthetic process, peptide metabolic process, protein metabolic process, and oxidation–reduction processes. These results suggest that Pb^2+^ stress alone markedly affected protein synthesis and redox-related cellular processes in TYL-A1. This response may reflect the increased demand for stress-response proteins, repair-related proteins, and redox regulation under Pb^2+^ exposure [43].

In the APbT vs. A comparison (Figure 5B), the enriched GO terms were more closely associated with membrane-associated components, transmembrane transport, transporter activity, ion transport, cation transport, oxidoreductase activity, and organic acid metabolism. This indicates that under combined Pb^2+^ + TYL stress, TYL-A1 may regulate membrane transport, ion homeostasis, and energy-related metabolic processes to adapt to combined stress. These findings are consistent with the observed changes in membrane permeability, cell surface hydrophobicity, ROS accumulation, and ATP levels. In the APbT vs. APb comparison (Figure 5C), the enriched terms were mainly associated with metal ion binding, cation binding, metal ion transport, ion transport, nucleic acid binding, DNA binding, proteolysis, membrane, and ribosome-related functions. These results indicate that the transcriptional response of TYL-A1 to TYL was relatively specific, mainly involving metal ion homeostasis, transcriptional regulation, protein turnover, and membrane-associated processes, rather than causing broad transcriptomic perturbation. Overall, the GO enrichment results indicate that TYL-A1 responded to Pb^2+^ and Pb^2+^ + TYL stress through coordinated regulation of protein synthesis, membrane transport, ion homeostasis, redox processes, and energy-related metabolism. These functional changes may contribute to the ability of TYL-A1 to maintain physiological stability and TYL removal capacity under co-contamination stress.

**Figure 6:**
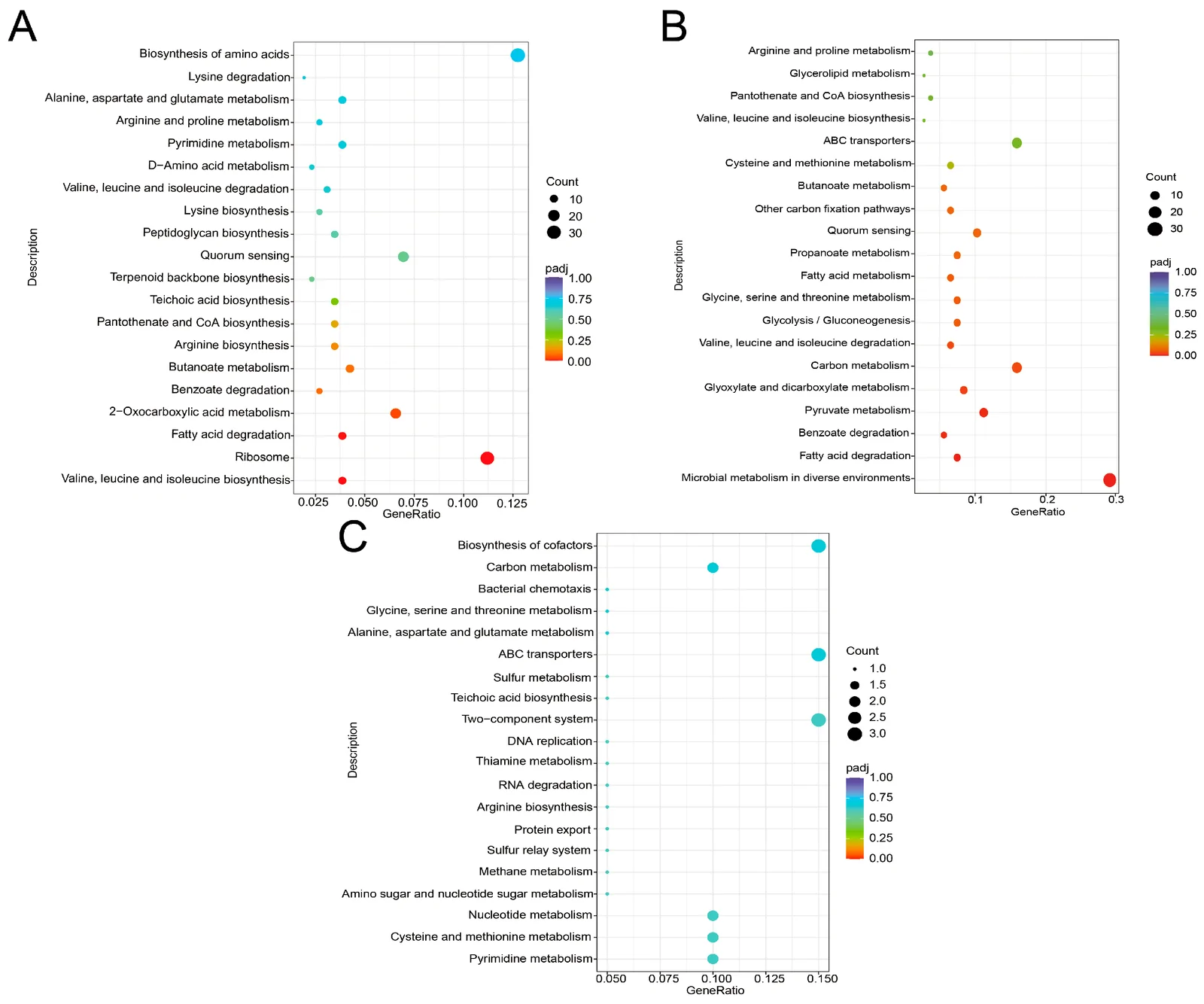
KEGG functional enrichment analysis of differentially expressed genes. (**A**) AP vs. A; (**B**) APbT vs. A; (**C**) APbT vs. APb.

KEGG pathway analysis revealed distinct metabolic responses of TYL-A1 under Pb^2+^ stress alone and combined Pb^2+^-TYL stress. In the AP vs. A comparison (Figure 6A), the ribosome pathway was significantly enriched, and associated differentially expressed genes were upregulated. This suggests that the cells may compensate for Pb^2+^-induced protein damage by enhancing ribosome-related functions and protein synthesis. Meanwhile, genes associated with valine, leucine and isoleucine biosynthesis, fatty acid degradation, and 2-oxocarboxylic acid metabolism were consistently downregulated, indicating that Pb^2+^ induced a protein synthesis-related response while suppressing certain biosynthetic and catabolic processes. This pattern may reflect the redistribution of cellular metabolic resources under stress conditions. In the APbT vs. A comparison (Figure 6B), the significantly enriched pathways mainly included microbial metabolism in diverse environments, carbon metabolism, pyruvate metabolism, glyoxylate and dicarboxylate metabolism, fatty acid degradation, benzoate degradation, and valine, leucine and isoleucine degradation. The differentially expressed genes in these pathways were associated with fatty acid degradation, benzoate degradation, glyoxylate and dicarboxylate metabolism, and branched-chain amino acid degradation. These results indicate that combined stress did not broadly enhance energy metabolism but instead suppressed multiple central metabolic and catabolic processes. This expression pattern may reflect a reduction in energy expenditure on substrate degradation and utilization under sustained combined stress, allowing the limited cellular resources to be preferentially allocated to the maintenance of basic physiological functions and stress adaptation. In the APbT vs. AP comparison (Figure 6C), differentially expressed genes were annotated to pathways including two-component systems, ABC transporters, carbon metabolism, and nucleotide metabolism. However, only a small number of genes were involved, and both upregulated and downregulated genes were present. Therefore, these changes are more likely to represent gene-specific responses induced by TYL under a Pb^2+^ stress background rather than the overall activation of the corresponding pathways.

### 3.6. Removal of TYL from Aquaculture Wastewater

**Figure 7:**
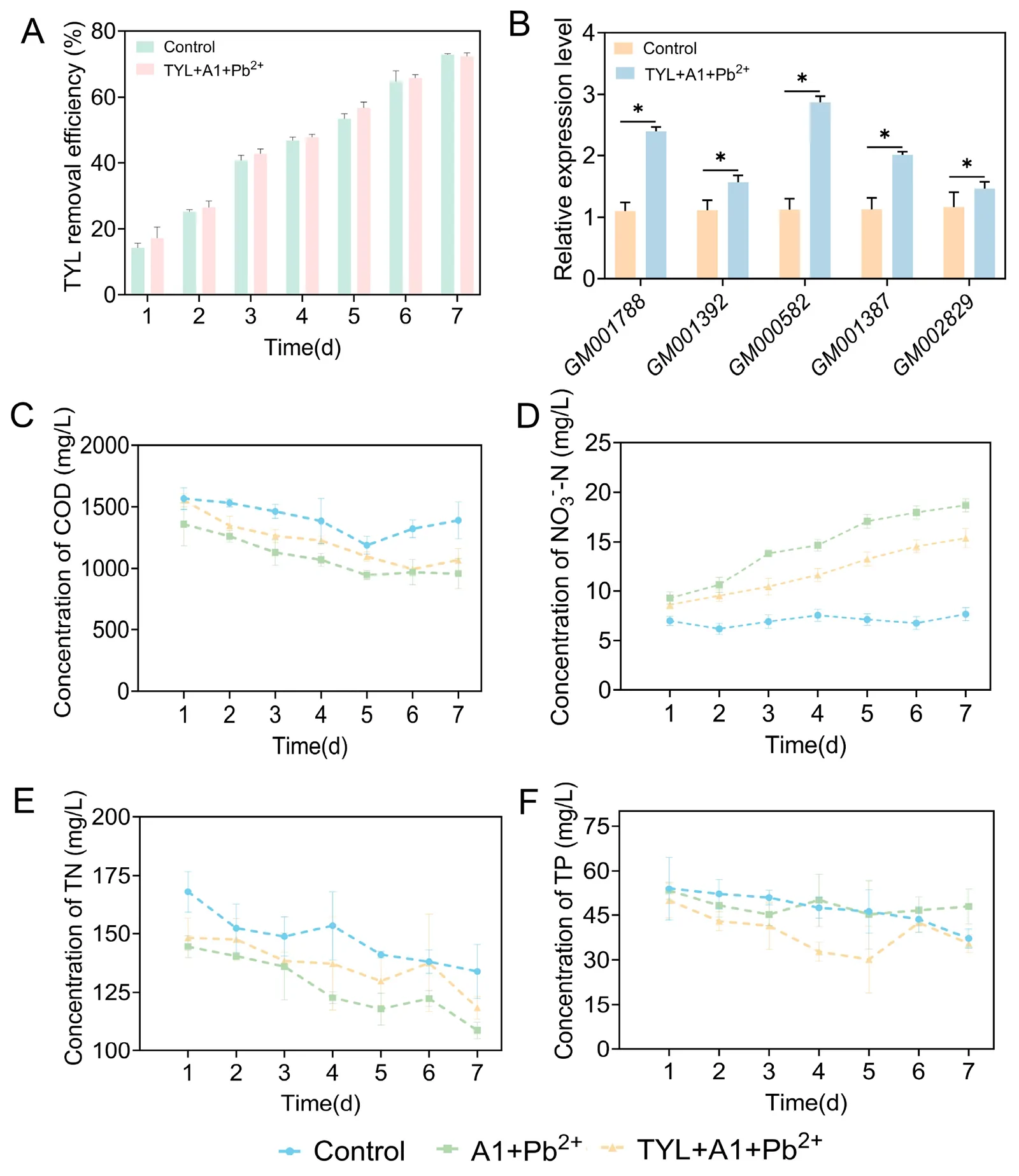
Removal of TYL in aquaculture wastewater using strain TYL-A1 and changes in water quality indicators under Pb^2+^ stress. (**A**) TYL removal efficiency; (**B**) RT-qPCR validation of five candidate stress-responsive genes; (**C**) COD concentration; (**D**) NO_3_^−^-N concentration; (**E**) TN concentration; (**F**) TP concentration. (* *p* < 0.05, compared to the control group).

As shown in Figure 7A, the concentration of TYL in the simulated aquaculture wastewater gradually decreased during incubation, with TYL removal exceeding 75% within 7 days. This result indicates that strain TYL-A1 retained the ability to remove TYL in the wastewater matrix. To further investigate the adaptive responses of TYL-A1 under simulated aquaculture wastewater and combined Pb^2+^ + TYL stress conditions, selected candidate stress-response genes were analyzed by quantitative real-time PCR. As shown in Figure 7B, *GM001788*, *GM000582*, *GM001387*, *GM001392*, and *GM002829* were all upregulated.

The expression patterns of candidate genes further supported this stress-adaptation interpretation. *GM001392* encodes a putative CheR-like methyltransferase and may participate in chemotactic sensory adaptation and environmental signal responses [44]. *GM001788* encodes CTP synthase and may contribute to maintaining the cellular CTP supply [45]. *GM001387* encodes the ATP-dependent RNA helicase DbpA and may participate in 23S rRNA maturation and 50S ribosomal subunit assembly [46], potentially reflecting a compensatory response to TYL-associated translational stress. *GM000582* encodes the high-affinity zinc uptake protein ZnuA and may contribute to zinc homeostasis and the maintenance of zinc-dependent enzyme functions [47,48]. These putative functions are consistent with the physiological pressures imposed by combined Pb^2+^ and TYL exposure. Pb^2+^ can induce oxidative stress and protein misfolding [49,50], whereas TYL inhibits protein synthesis and may impair membrane integrity, potentially resulting in growth inhibition and metabolic disturbance. Accordingly, the upregulation of these candidate genes may reflect compensatory regulation that helps TYL-A1 maintain cellular viability and essential metabolic functions under combined stress. *GM002829* encodes an α/β hydrolase whose ability to remove TYL was previously demonstrated [23]. In the present study, both transcriptomic analysis and RT-qPCR confirmed its upregulation under combined Pb^2+^ + TYL exposure. This result, together with the maintained TYL removal performance, indicates that the previously identified TYL-degradation gene remained actively expressed under the tested combined-stress condition. Overall, the concurrent upregulation of these genes may help maintain cellular viability and basal metabolic activity, thereby indirectly supporting TYL removal. However, these interpretations are primarily based on functional annotations and transcriptional correlations and do not establish direct causal relationships with stress adaptation or TYL removal. Therefore, further validation through gene knockout, complementation, and enzymatic assays is required.

Based on these findings, the application potential of strain TYL-A1 in aquaculture wastewater underwent further evaluation. The abundant and compositionally diverse organic matter in wastewater creates a multi-substrate environment [51,52,53], in which readily bioavailable organic fractions may be preferentially utilized. This preferential substrate utilization may induce carbon catabolite repression or redirect cellular metabolic resources away from TYL removal [54]. The lower TYL removal observed in the simulated wastewater than in the MSM system is consistent with this possibility, suggesting that the utilization of complex organic fractions may have diverted metabolic resources away from TYL transformation and reduced the overall TYL removal efficiency. However, the relative contributions of carbon catabolite repression, metabolic resource competition, and other wastewater matrix effects require further investigation.

COD is a key indicator of the total amount of oxidizable organic matter in water [55]. In this study, COD decreased during TYL removal (Figure 7C), suggesting that strain TYL-A1 may promote the transformation of some oxidizable organic matter in wastewater while removing the target antibiotic. However, because no metabolite identification and mineralization assessments were performed, it remains unclear whether TYL was completely mineralized or merely transformed into potentially persistent intermediates. Further LC–MS analysis and mineralization assays are required to clarify its transformation pathway and degree of mineralization. As shown in Figure 7D, the NO_3_^−^-N content was higher in the Pb^2+^ and Pb^2+^ + TYL treatment groups than in the control group. This increase may reflect the transformation of other nitrogen species into nitrate during incubation. However, the specific nitrogen transformation pathways require further confirmation through analyses of NO_2_^−^-N, NH_4_^+^-N, and related functional genes. Eutrophication is one of the major global environmental issues, and phosphorus pollution is a major driving factor [56]. Excessive phosphorus in water can disrupt aquatic ecological balance and threaten water resources and human health [57]. In the Pb^2+^ single-stress group, TP showed only minor differences compared with the control group (Figure 7F). In contrast, TP in the combined-stress group decreased significantly from day 3 to day 5 and then gradually increased on day 6. This decrease may be partly attributed to phosphorus assimilation by TYL-A1 during bacterial growth and stress adaptation. Phosphorus availability may enhance bacterial metabolic activity and indirectly promote TYL degradation. However, in the presence of Pb^2+^, other physicochemical processes may also contribute to the decrease in aqueous TP, including phosphate precipitation with metal ions and the adsorption of phosphorus onto bacterial cells, extracellular polymeric substances, or suspended particles [26]. The subsequent increase in TP may be related to the release of intracellular phosphorus due to cell damage or lysis, the desorption of previously bound phosphorus, or changes in the precipitation–dissolution equilibrium [58]. These observations are consistent with previous findings indicating that microorganisms can utilize exogenous phosphorus to enhance the degradation of organic pollutants and mitigate environmental toxicity, thereby providing a theoretical basis for nutrient-regulation-based water remediation strategies [59,60]. TN is commonly used as an indicator of nitrogen pollution in water bodies. The TN concentrations in both the Pb^2+^ and Pb^2+^+TYL groups remained lower than those in the control group (Figure 7E), suggesting that the assimilation and utilization of nitrogen during bacterial growth and adaptation to stress may be one of the reasons for the decline in TN. Meanwhile, the increase in NO_3_^−^-N suggests that transformations among different nitrogen species may also have occurred in the system. Furthermore, potential gaseous losses through denitrification or ammonia volatilization cannot be ruled out. Because a complete mass-balance analysis was not performed, the relative contributions of microbial assimilation, nitrogen transformation, gaseous loss, adsorption, and precipitation could not be quantified.

Taken together, the physiological, biochemical, and transcriptomic results suggest that combined Pb^2+^ + TYL stress induced oxidative stress, membrane-associated alterations, pressure on energy metabolism, and transcriptional changes in TYL-A1. Oxidative stress responses, membrane-related changes, transport-associated pathways, translation-related processes, and central carbon metabolism were associated with the adaptation of TYL-A1 under the tested conditions. These responses may help explain how TYL-A1 maintained TYL removal performance under a nominal Pb^2+^ addition in the phosphate-containing MSM system. However, the direct causal links between specific pathways and TYL removal efficiency remain to be established through further functional validation. The mechanistic analysis in this study was mainly based on correlations between physiological and biochemical indicators and transcriptomic data. Therefore, the relevant conclusions remain mechanistic inferences and still require further validation through approaches such as gene knockout, protein expression analysis, metabolite detection, and enzyme activity assays to clarify the specific roles of the key pathways. Because the medium contained KH_2_PO_4_ and K_2_HPO_4_, Pb^2+^ may have undergone phosphate-mediated precipitation or complexation, thereby reducing the dissolved and bioavailable Pb^2+^ fraction. Therefore, the added Pb^2+^ concentration of 100 mg/L should be regarded as a nominal concentration rather than the actual concentration of freely available Pb^2+^. Accordingly, the observed physiological and transcriptional responses of strain TYL-A1 should be interpreted within the context of the current phosphate-containing culture system and should not be directly taken to represent its tolerance under fully bioavailable Pb^2+^ exposure. Future studies should include growth and TYL removal controls using low-complexing, metal-compatible buffer systems, such as PIPES or HEPES, together with measurements of dissolved or free Pb^2+^, to better evaluate the actual Pb^2+^ toxicity and tolerance threshold of TYL-A1.

In addition, the aquaculture wastewater used in this study was filtered, diluted and autoclaved before use. These treatments reduced background interference and allowed the TYL removal ability of TYL-A1 to be evaluated under controlled laboratory conditions. However, they also simplified the wastewater matrix and removed the native microbial community. Therefore, the wastewater assay should be regarded as a preliminary proof-of-concept test rather than direct evidence of its performance in raw, untreated wastewater. Under field-relevant conditions, TYL-A1 would need to tolerate higher organic loads, more complex physicochemical conditions, and competition from indigenous microorganisms. Further studies using non-sterilized, less diluted, and ultimately raw farm wastewater are required to provide a more reliable assessment of its practical application potential.

## 4. Conclusions

This study provides physiological and transcriptomic insights into the maintenance of TYL removal using strain TYL-A1 under nominal Pb^2+^ exposure in a phosphate-containing culture system. After 72 h of cultivation in phosphate-containing MSM supplemented with a nominal Pb^2+^ concentration of 100 mg/L, TYL removal remained above 85%, indicating that TYL-A1 retained substantial removal capacity under the tested conditions. Pb^2+^ stress increased SOD and CAT activities while also causing elevated levels of ROS and MDA, along with a decline in ATP content, indicating that the strain was subjected to pronounced oxidative stress and energy metabolic pressure. Changes in membrane permeability and cell surface hydrophobicity, together with SEM and FTIR results, suggested that TYL-A1 may enhance its environmental adaptability through membrane structure remodeling. Transcriptomic analysis showed that pathways related to ABC transporters, two-component systems, carbon metabolism, and nucleotide metabolism pathways were involved in its adaptive response. These findings provide preliminary proof-of-concept evidence for the potential application of TYL-A1 in antibiotic–heavy metal co-contaminated environments. However, because the wastewater was filtered, diluted, and autoclaved, these results should not be considered as robust evidence of its efficacy in raw, untreated farm wastewater. Further validation in non-sterilized, less diluted, and more complex wastewater systems is therefore required.

## Data Availability

The data that support the findings of this study are available from the corresponding author on a reasonable request. The raw sequencing data generated in this study have been deposited in the NCBI database under BioProject accession number PRJNA1457065.

## Supplementary Materials

The following supporting information can be downloaded at: https://www.mdpi.com/article/10.3390/antiox15080958/s1, Figure S1: TYL removal in abiotic (AC), heat-inactivated cell (HKC), and live-cell treatments under Pb^2+^ stress; Figure S2: Volcano plots of differentially expressed genes in strain TYL-A1 under Pb^2+^ or Pb^2+^ + TYL stress. A: APb vs. A; B: APbT vs. A; C: APbT vs. APb; Table S1: Lead concentrations in culture supernatants after centrifugation and 0.45 μm filtration. (APb, TYL-A1 + Pb^2+^; APbT, TYL-A1 + Pb^2+^ + TYL; PbT, Pb^2+^ + TYL; Pb, Pb only).
