# Supplementary material for: Tylosin Removal Under Pb2+ Stress Using Kurthia gibsonii TYL-A1: Redox Adaptation and Transcriptomic Insights
Source: Antioxidants (Basel). 2026 Jul 31;15(8):958. doi: 10.3390/antiox15080958 (PMC13509506; doi:10.3390/antiox15080958)
Supplement: Supplementary file 1 [file antioxidants-15-00958-s001.zip › antioxidants-4452027-supplementary.pdf]

Tylosin Removal Under Pb^{2+} Stress Using *Kurthia gibsonii* TYL-A1: Redox Adaptation and Transcriptomic Insights

Ye Wang [1], Heshi Tian [1], Xiqing Zhang [2], Yuanquan Zhu [1], Lingcong Kong [1], Changlong Gou [3], Hongxia Ma [1], Xiuzhen Yu [4], Wenyan Yang [1,*] and Yunhang Gao [1,*]

[1] College of Veterinary Medicine, Jilin Agricultural University, Changchun 130118, China
[2] Northeast Agricultural Research Center of China, Jilin Academy of Agricultural Sciences, Changchun 130033, China
[3] College of Animal Science and Technology, Inner Mongolia Minzu University, Tongliao 028000, China
[4] Institute of Agricultural Equipment, Xinjiang Academy of Agricultural Sciences, Urumqi 830091, China
* Correspondence: yangwy760913@163.com (W.Y.); gaoyunhang@163.com (Y.G.)

Treatment	0 d (mg/L)	1 d (mg/L)	3 d (mg/L)
APb	3.90	1.05	1.00
APbT	2.70	1.00	0.90
PbT	2.90	1.80	1.90
Pb	4.47	3.05	2.90

Table S1. Lead concentrations in culture supernatants after centrifugation and 0.45 μm filtration. (APb, TYL-A1 + Pb^{2+}; APbT, TYL-A1 + Pb^{2+} + TYL; PbT, Pb^{2+} + TYL; Pb, Pb only)

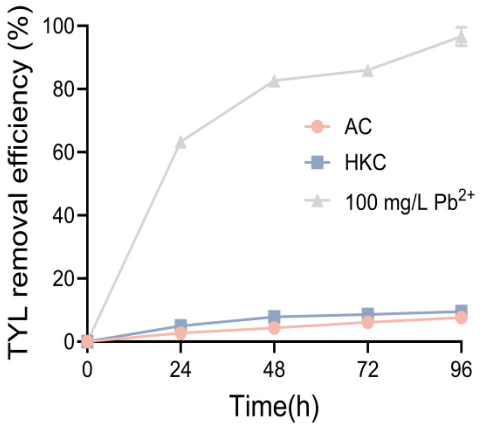

Figure S1. TYL removal in abiotic (AC), heat-inactivated cell(HKC), and live-cell treatments under Pb^{2+} stress.

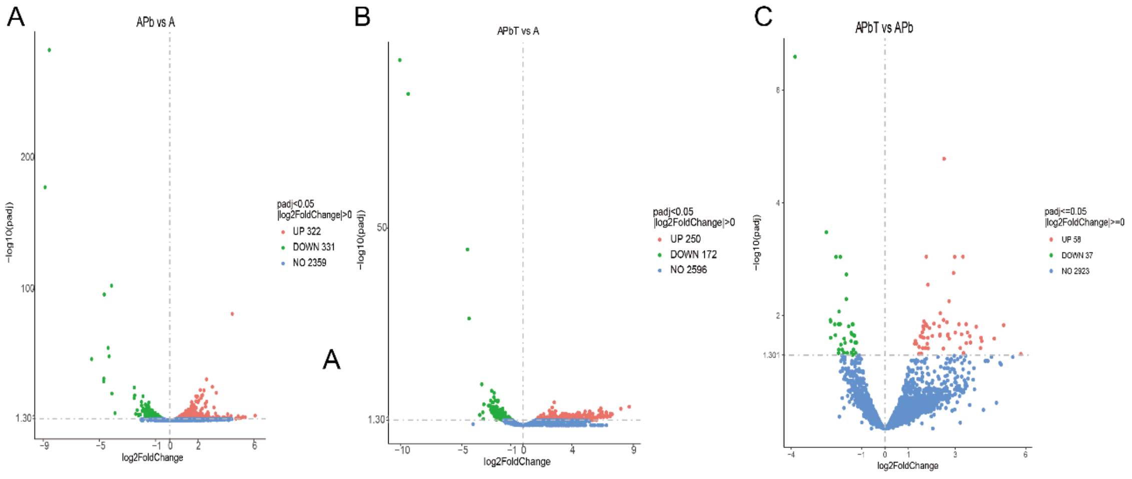

Figure S2. Volcano plots of differentially expressed genes in strain TYL-A1 under Pb^{2+} or Pb^{2+} + TYL stress. A: APb vs. A; B: APbT vs. A; C: APbT vs. APb
